# Late-onset mitochondrial encephalomyopathy lactic acidosis and stroke-like episodes with chronic intestinal pseudo-obstruction: a case-based review

**DOI:** 10.3389/fmolb.2026.1857438

**Published:** 2026-08-14

**Authors:** Lin Zhang, Junjian Lin, Fengying Li, Yujie Jia, Junhua Li, Xiaopeng Wang

**Affiliations:** 1 Department of Neurology, Rizhao Central Hospital, Rizhao, China; 2 Department of Rehabilitation Medicine, Rizhao Central Hospital, Rizhao, China; 3 Department of Neurology, The Second Hospital of Hebei Medical University, Shijiazhuang, China

**Keywords:** mitochondrial encephalomyopathy, mitochondrial encephalomyopathy lactic acidosis and stroke-like episodes, chronic intestinal pseudo-obstruction, m.3243A>G gene mutation, late-onset

## Abstract

**Background:**

Mitochondrial Encephalomyopathy Lactic Acidosis and Stroke-like episodes (MELAS) is rare in people over 40 years of age, and gastrointestinal complications of MELAS are also rare, especially Chronic Intestinal Pseudo-Obstruction (CIPO).

**Case Presentation:**

This report describes a 60 years old MELAS patient with a mutation rate of only 6.29% in the m.3243A>G gene, accompanied by CIPO. After treatment, MELAS symptoms can be controlled, but intestinal obstruction recurs and worsens. At present, patients fast and rely on intravenous nutrition to sustain their lives.

**Discussion:**

The low proportion of m.3243A>G mutations in patients may be related to their advanced age, but the high or low proportion of gene mutations detected in blood samples is not related to the severity of symptoms. MELAS combined with CIPO is rare and different from other intestinal obstructions. CIPO has no cause of mechanical intestinal obstruction and may be related to dysfunction of smooth muscle mitochondria or involvement of the enteric nervous system.

**Conclusion:**

For complications of MELAS beyond the nervous system, early consideration should be given to the possibility of MELAS. Early genetic testing has important clinical significance for the treatment and prognosis of patients. This article will provide a literature review on the multi system performance of MELAS.

## 1 Introduction

Mitochondrial DNA mutations exceeding the critical threshold of disease can lead to mitochondrial disease (MD) (Wang et al., 2020). MELAS is the most common mitochondrial encephalomyopathy (ME) (Wu et al., 2021), it most commonly occurs in the population aged 2–31 years and is very rare after the age of 40 years. Eighty percent of MELAS cases are caused by mitochondrial DNA 343A>G mutations (Wu et al., 2021). Although the mtDNA A3243G mutation can be detected in white blood cells, the mutation level decreases over time (Fan et al., 2021). MELAS is a multisystem metabolic disorder (Wang et al., 2020), including neurological manifestations such as stroke, headache, cognitive impairment, epileptic seizures, partially reversible aphasia, cortical vision loss, and motor weakness, as well as rare gastrointestinal manifestations (Chinese Medical Association Neurology Branch, 2015; Mancuso et al., 2014; Wang et al., 2008), and in severe cases, CIPO (Narbonne et al., 2004; De Laat et al., 2015; Sproule and Kaufmann, 2008). CIPO is a rare chronic intestinal obstruction syndrome characterized by episodic exacerbation (Jinnouchi et al., 2022). This article reports a rare case of late-onset MELAS with recurrent CIPO, and further research is needed on the mechanism by which MELAS causes CIPO. A comprehensive understanding of the various systemic complications of MELAS is necessary in clinical practice.

## 2 Case report

### 2.1 Performance of MELAS

This patient is 60 years old. He was admitted on 13 November 2022, because of bilateral visual blurring. Physical examination revealed short stature (height 158 cm) and low body weight (40 kg). Further neurological evaluation revealed profound bilateral sensorineural hearing loss and right homonymous hemianopia. Brain MRI-Diffusion-Weighted-Imaging (DWI) revealed acute cerebral infarction in the left temporal parietal occipital lobe (Lace sign). No stenosis was detected in the basal artery, left vertebral artery, or left posterior cerebral artery during brain MRI-Magnetic Resonance Angiography (MRA), and the infarct area did not conform to vascular distribution.

Past history: Progressive binaural hearing loss since 2007 and type 2 diabetes diagnosed in May 2022. Gastroscopy examination on 5 August 2022 diagnosed gastric mucosal erosion and chronic non atrophic gastritis. History of chronic abdominal distension for many years.

Family history: His son is also short in stature, with a height of 165 cm. His mother had a history of mental disorders, which appeared at a young age and worsened in middle age.

After receiving antiplatelet therapy, lipid-lowering medication, plaque-stabilizing treatment, and circulation-enhancing therapy, his condition initially improved. On 21 November 2022, the family discovered that the patient had slow reactions, could not recognize their family members, and could not take care of themselves. Neurological examination suggests that the patient’s computational, directional, memory, and executive abilities have all decreased. Re-examination via brain MRI-DWI revealed left occipital parietal lobe cerebral infarction (Lace sign). The patient experienced cerebral infarction again, but no cerebral artery stenosis was found through brain magnetic resonance imaging MRA. Similarly, the infarcted area does not conform to the distribution of blood vessels. From 22 November 2022 to 24 November 2022, the patient repeatedly experienced epileptic seizures, and the symptoms improved after treatment with levetiracetam. On 23 November 2022, the patient experienced abnormal mental behavior, irritability, and aggressive behavior. After treatment with diazepam and olanzapine, the condition improved.

#### 2.1.1 Meaningful imaging examinations

**FIGURE 1:**
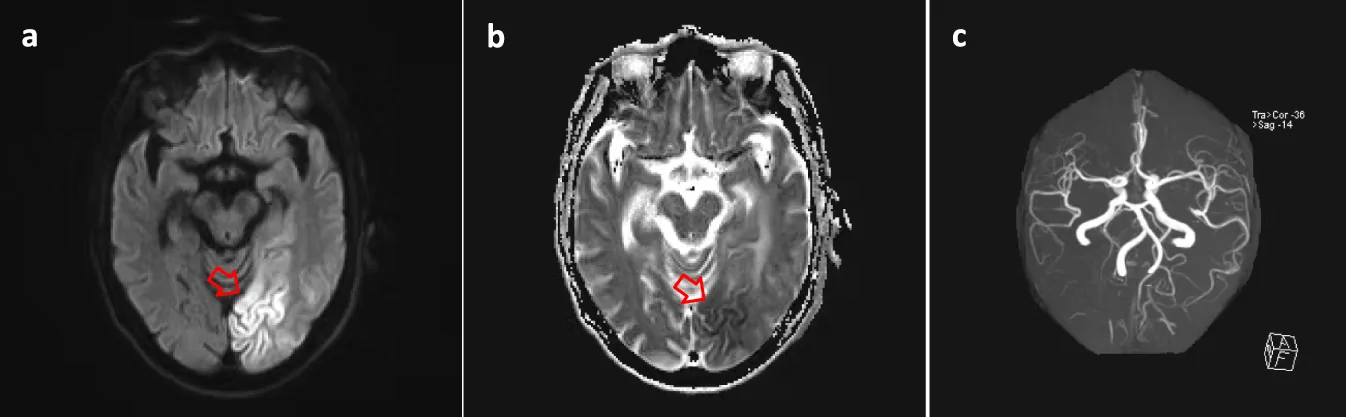
13 November 2022 MRI: **(a,b)** Lacey like DWI high signal and ADC low signal are observed in the left temporal occipital parietal lobe; **(c)** MRA did not show cerebral artery stenosis in the lesion area, which does not conform to vascular distribution.

On 13 November 2022, brain MRI-DWI\Apparent Diffusion Coefficient (ADC) \MRA (Figure 1) showed acute cerebral infarction in the left temporal occipital parietal lobe and no obvious arterial stenosis.

**FIGURE 2:**
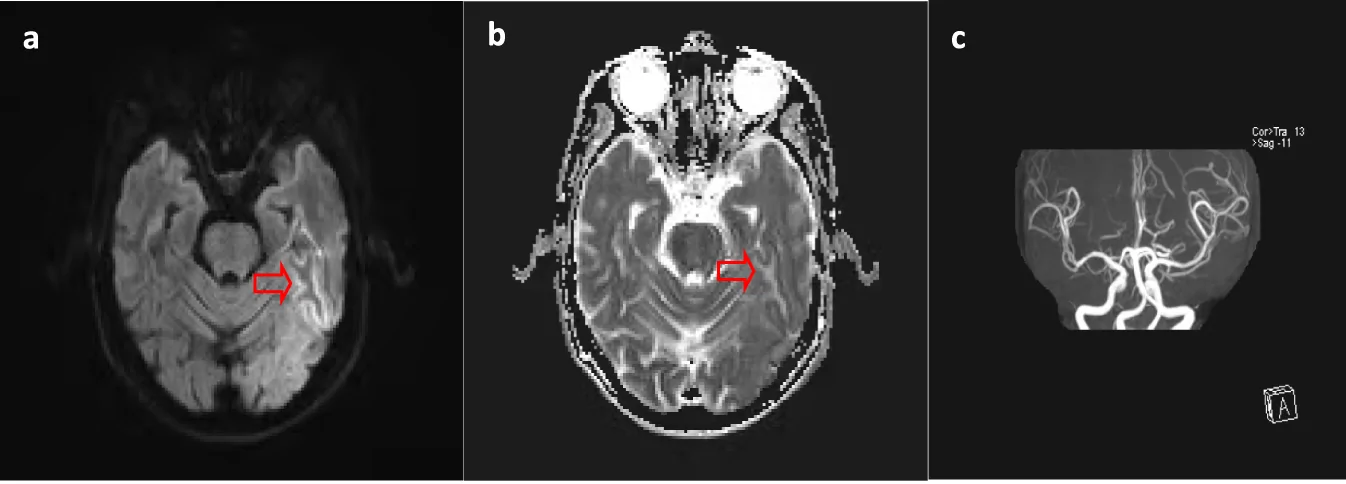
21 November 2022 MRI: **(a,b)** Lacey like DWI high signal and ADC low signal are observed in the left temporal occipital parietal lobe; **(c)** MRA did not show cerebral artery stenosis in the lesion area, which does not conform to vascular distribution.

On 21 November 2022, brain MRI-DWI\ADC\MRA (Figure 2) revealed a left occipital parietal lobe cerebral infarction (with obvious border signs compared with the previous MRI).

**FIGURE 3:**
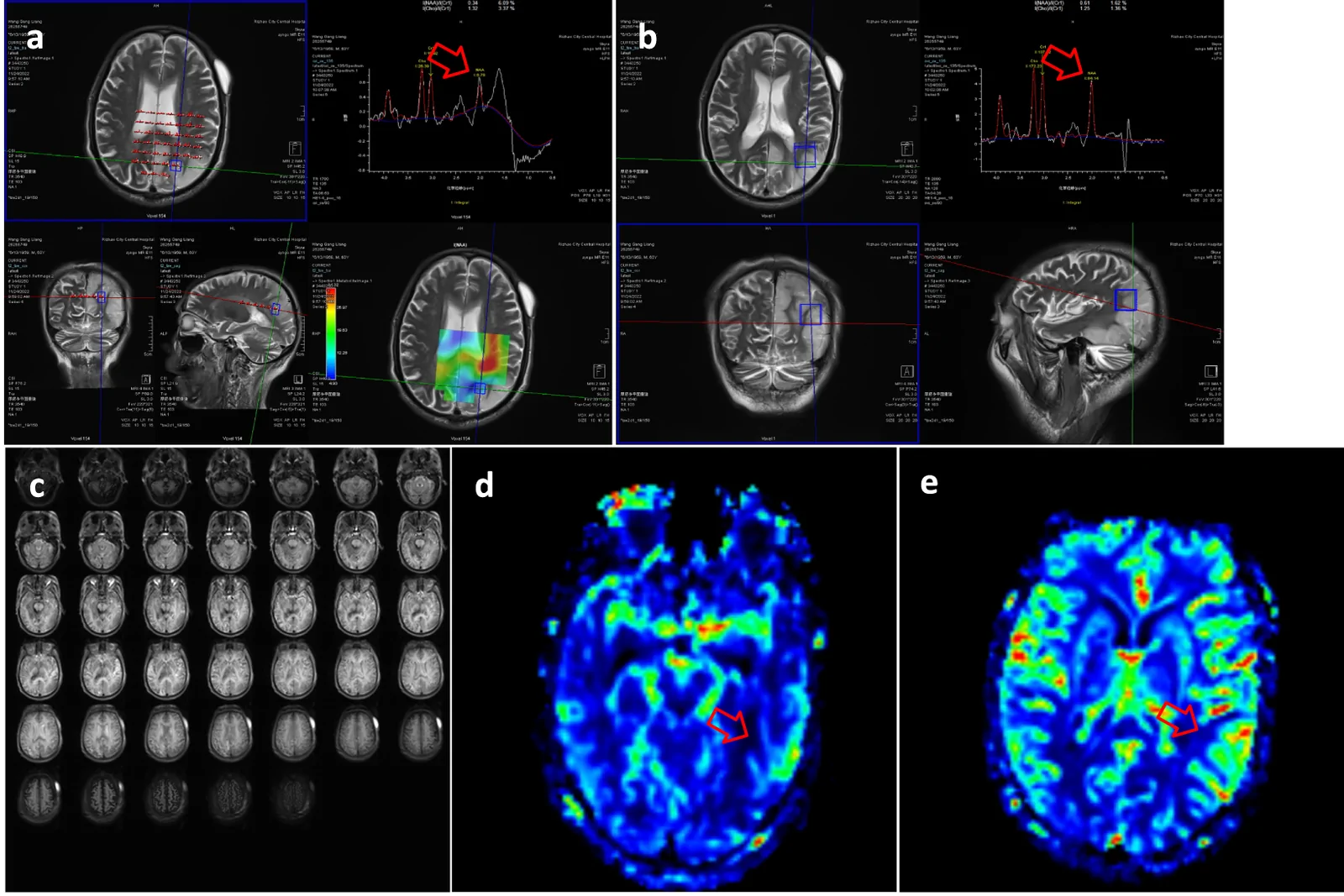
24 November 2022: **(a,b)** MRS (lesion\CSF) showed a decrease in N-acetylaspartate (NAA), and Lactic acid (LAC) peak was visible. **(c–e)** ASL shows high perfusion at the lesion site.

On 24 November 2022, brain MRI-Magnetic Resonance Spectroscopy (MRS) (lesion\Cerebrospinal fluid, CSF), enhancement, perfusion, MRI-Arterial Spin Labeling (ASL) (Figure 3): Linear enhancement was observed on the surface of some cerebral gyri in the low-signal area of the left temporal occipital lobe on T1-weighted imaging (T1WI), with many blood vessels running around it. MRI-MRS shows a lactate peak. The MRI-ASL lesion area shows high perfusion.

On 22 November 2022, Video-Electroencephalogram (EEG): Abnormal EEG, with sleep disturbances and roughly normal sleep cycles. Slow wave: Diffuse 5–7 Hz moderate amplitude slow wave activity during wakefulness that is partially rhythmic.

On 30 November 2022, Color Doppler ultrasound of lymph nodes: No abnormal enlarged lymph node echoes were detected in the bilateral inguinal regions or bilateral axillary areas. No abnormal enlarged lymph node echoes were detected in the bilateral cervical regions or bilateral supraclavicular fossae.

On 1 December 2022, Chest CT: Ultiple nodules in the upper lobe of both lungs and the lower lobe of the right lung, with the largest one located in the upper lobe of the left lung, Im(107–112,1 mm), The size is approximately 4 × 4 mm.

On 29 April 2023, Color Doppler ultrasound of lymph nodes: No abnormal enlarged lymph node echoes were detected in both armpits.

Mini-Mental State Examination (MMSE) (junior high school dropout):7/30 points.

#### 2.1.2 Meaningful laboratory tests

On 14 November 2022, the blood glucose level was 6.9 mmol/L (3.9–6.1 mmol/L).

On 22 November 2022, glycated hemoglobin 7.4% (4.5%–6.5%).

On 24 November 2022, the serum lactic acid (resting) concentration was 2.2 mmol/L (0.7–2.1 mmol/L), and the serum lactic acid (after activity) concentration was 3.6 mmol/L (0.7–2.1 mmol/L).

On 25 November 2022, autoimmune encephalitis related antibody spectrum (serum\CSF): Anti Tr antibody (DNER): 1:30.

On November 24–30, 2022, the CFS examination revealed the following results: the CSF glucose concentration was 5.5 mmol/L (2.5–4.4 mmol/L), and the CSF protein concentration was 578.00 mg/L (200–400 mg/L). CSF IgG was 71.4 mg/L (10.3–30.0 mg/L).

On 15 December 2022, a serum MT-TL1 gene examination revealed the m.3243A>G mutation, with a mutation rate of 6.29% (American College of Medical Genetics and Genomics, Classification: Pathogenic).

According to the findings of stroke-like seizures in the patient with hemianopia, head MRI-DWI revealed high signal intensity (flower border sign) in the temporal parietal occipital junction area, high perfusion in the MRI-ASL lesion area, inconsistency with the large blood vessel supply area, and abnormal blood lactate exercise test results: lactate did not decrease after exercise, m.3243A>G gene mutation, MRS showed a lactate peak, neurogenic hearing loss, short stature, recurrent epilepsy, and decreased intelligence, which can confirm MELAS. MELAS mostly occurs in the population aged 2–31 years old, and this patient developed the disease at the age of 60, which is a rare late-onset case that may be related to the low proportion of m.3243A>G gene mutations.

Treatment: Intravenous injection of arginine hydrochloride (20 mL qd) and lipoic acid (300 mg qd), oral administration of vitamin B1 tablets (10 mg tid), vitamin B2 tablets (5 mg tid), folic acid tablets (0.8 mg qd), vitamin B6 tablets (10 mg qd), methylcobalamin tablets (0.5 mg tid), coenzyme Q10 tablets (10 mg tid), oral administration of saxagliptin tablets (5 mg qd) to lower blood sugar, levetiracetam tablets (0.5 g bid) to control epilepsy, and olanzapine tablets (5 mg qd) to control psychiatric symptoms. After treatment, patients no longer exhibit aggressive behavior or epileptic seizures, and their blood sugar levels meet the standards. However, symptoms such as delayed response, intellectual decline, gait delay, hearing loss, and inability to communicate normally persist.

### 2.2 Performance of CIPO

**FIGURE 4:**
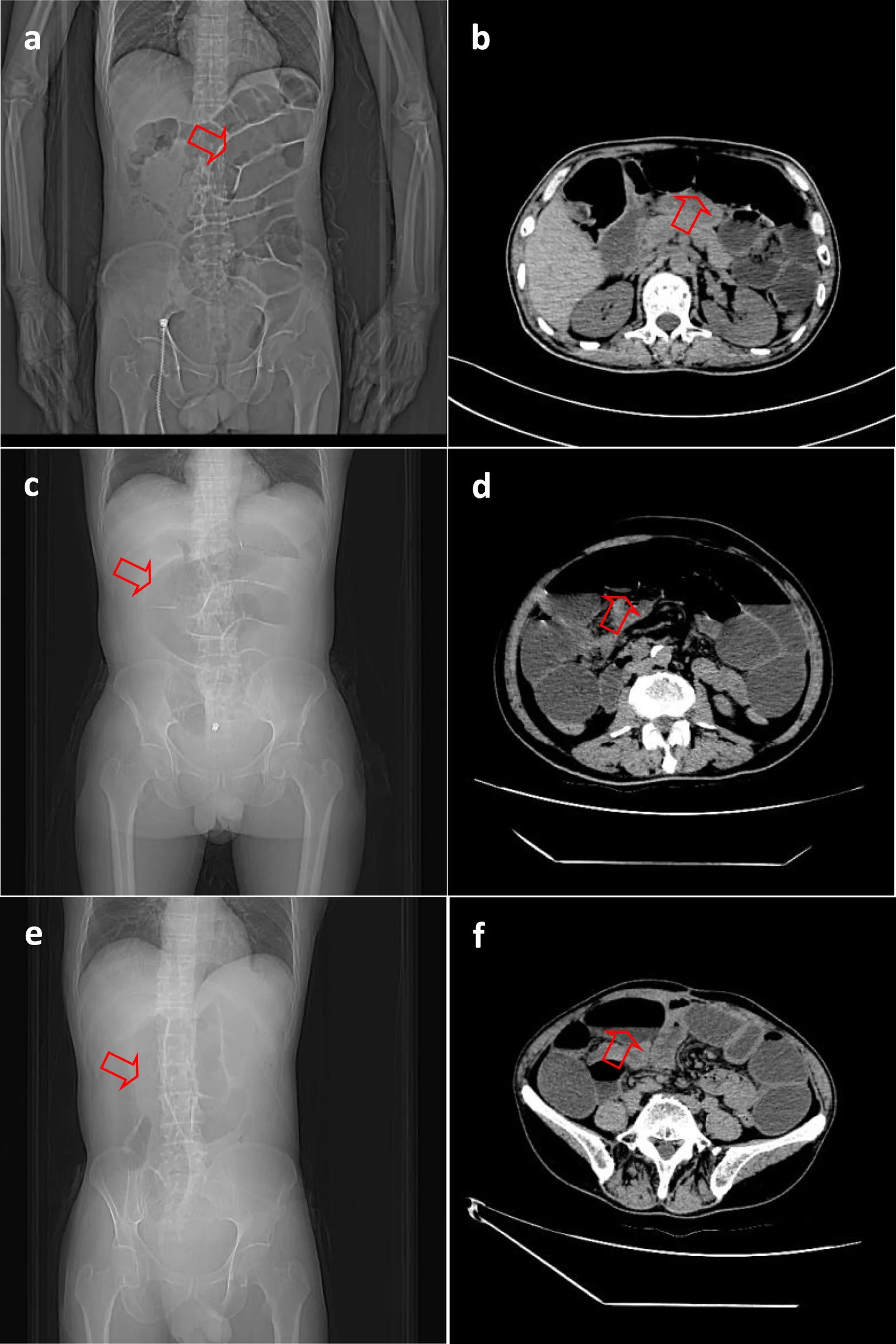
**(a,b)** CT, 30 November 2022. Abdominal intestinal dilation and gas accumulation, with multiple small fluid planes visible within the small intestine; **(c,d)** CT, 10 April 2024, Vortex sign is observed in the distal segment of the ileum and surrounding mesentery, with fluid accumulation and dilation in the upstream small intestine and visible gas-liquid plane; **(e,f)** CT, 6 February 2025. The mesenteric blood vessels show vortex like changes, and the small intestinal tract is dilated with fluid accumulation and a wide gas-liquid plane can be seen.

On 28 November 2022, the patient complained of severe abdominal distension. Abdominal Computed Tomography (CT) revealed intestinal obstruction. (Figures 4a,b). After open abdominal exploration, small intestine torsion was found, and the symptoms improved after surgery to reposition the small intestine. At present, the intestinal obstruction of patients is considered to be the intestinal motility disorder during the acute attack of MELAS.

On 8 April 2024, the patient experienced abdominal pain and bloating, accompanied by nausea and vomiting. Low exhaust, gradually worsening abdominal pain, physical examination shows bloating, tenderness throughout the abdomen, and weakened bowel sounds. On 10 April 2024, an abdominal CT (Figures 4c,d) revealed the following: small intestine obstruction.

From 22 December 2024 to 6 February 2025, the patient repeatedly experienced intestinal obstruction. On 6 February 2025, abdominal CT (Figures 4e,f) revealed a small intestinal obstruction, vortex-like changes in mesenteric blood vessels.

Follow-up: Currently, the frequency of patients experiencing persistent abdominal distension, constipation, and intestinal obstruction is increasing. Many times, patients rely on fasting and receiving intravenous nutrition to sustain their lives. The patient’s weight continues to decrease, and the patient is currently weighing approximately 35 kg.

From 2024 to now, the patient has recurrent abdominal distension and constipation. Abdominal CT showed intestinal obstruction with small bowel dilatation, but mechanical intestinal obstruction. The patient is considered to be MELAS combined with CIPO.

## 3 Discussion and review

The clinical characteristics, pathogenesis and treatment of MD, ME, MELAS and CIPO will be discussed below, and the multi system complications of me will be reviewed.

### 3.1 Mitochondria and MD

Mitochondria, as the energy factories of cells, are organelles with double-layered membranes in eukaryotic cells. This genetic system includes 13 messenger RNA genes, corresponding to the subunits of oxidative phosphorylation complexes, as well as mitochondrial transport RNA and ribosomal RNA. Mitochondrial DNA is prone to genetic mutations. If the proportion of mutated mitochondrial DNA exceeds the critical threshold of the disease, it may lead to gene expression and clinical symptoms. Mitochondrial DNA is only inherited through maternal lineage (Wang et al., 2020). MD has significant clinical heterogeneity in clinical manifestations and is more likely to involve multiple systems, with the brain and muscles, which require high energy, being the most susceptible. The genotype and clinical phenotype do not correspond one-to-one. Mitochondrial myopathy and cerebral myopathy are caused by pathogenic mutations in mitochondrial or nuclear genes, which in turn lead to damage to mitochondrial structure and function. These abnormalities can cause insufficient adenosine triphosphate production in skeletal muscle fibres and brain neurons, leading to muscle and/or brain lesions, manifested as muscle fatigue after activity, improvement after rest, and broken red fibres (Ragged red fibres, RRF) visible in muscle enzyme immunohistochemical staining (Wu et al., 2019).

### 3.2 MELAS and mitochondrial DNA 343A>G mutations

MELAS is the most common ME (Wu et al., 2021) and most commonly occurs in the population aged 2–31 years. It rarely develops after the age of 40, and existing reports in the literature focus mostly on pediatric or young patients. This case involved a rare case of late-onset MELAS. Eighty percent of MELAS cases are caused by mitochondrial DNA 343A>G mutations (Wu et al., 2021). Detecting mitochondrial DNA and nuclear DNA genes related to phenotype is crucial in diagnosing MELAS. Quantifying the proportion of mtDNA carrying wild-type and pathogenic mutations is crucial for understanding the disease progression of MD and evaluating the effectiveness of treatment methods. White blood cells, hair follicles, urine sediment, cheek mucosa, saliva, and skeletal muscle tissue can all be used for diagnostic testing. Although the mtDNA A3243G mutation can be detected in white blood cells, the mutation level decreases over time, resulting in very low or undetectable mutation levels in patients with severe MELAS syndrome. Research has shown that blood sample analysis is not helpful in predicting the prognosis of patients with MELAS syndrome, as there is no significant correlation between the mutation burden in the blood and the clinical characteristics of patients. In addition, the mtDNA A3243G mutation burden in the blood may not be a good indicator of the overall mutation burden in affected tissues (Fan et al., 2021), so the proportion of m.3243A>G mutations in this patient was only 6.29%, which may be related to the patient undergoing genetic testing at the age of 60. Although the mutation rate of the patient is low, the severity of the patient’s symptoms may be related to the blood sample chosen for testing.

### 3.3 Clinical manifestations of ME

ME is a multi system metabolic disease (Wang et al., 2020). Stroke such as seizures are the most typical clinical manifestation of the MELAS nervous system, with other manifestations including headache, cognitive impairment, epileptic seizures, partially reversible aphasia, cortical visual loss, and motor weakness. MRI reveals almost normal or stroke-like attacks, cortical atrophy, white matter lesions, and underdeveloped or underdeveloped corpus callosa. MRS revealed an increase in the N-acetylaspartate and LAC peaks. Asymmetric infarction may occur in the affected area, and DWI often shows diffusion limitation, with cortical involvement being particularly prominent, often presenting as gyrus like hyperintensities, namely lace or ribbon signs. The temporal lobe, parietal lobe, and occipital lobe do not conform to the distribution of the watershed area of a single intracranial artery. The damage may be limited to the cortex or subcortical white matter (Sun and Weidong, 2020). Approximately 40%–90% of MELAS syndrome patients experience dementia, and 71%–96% experience epilepsy (systemic or focal seizures). The peripheral nervous system is characterized by axonal or mixed axonal and demyelinating neuropathy according to electrophysiological studies. Some patients may have fever and headache. The manifestations of mental state changes include anxiety, bipolar disorder, depression, mental illness, and personality changes. Ophthalmic manifestations include ophthalmoplegia optic nerve atrophy (Perez-Atayden, 2013) and pigmentary retinopathy. MELAS patients may have hearing problems, including early-onset, mild, and progressive sensorineural hearing loss, as well as peripheral neuropathy associated with chronic and progressive hearing loss (Tang et al., 2014). ME patients may experience symptoms of cardiomyopathy. A total of 21%–33% of ME patients have type 1 or type 2 diabetes. Energy deficiency caused by mitochondrial mutation leads to impaired insulin secretion and insulin reduction. The short stature of ME patients may be due to long-term energy deficiency. Occasionally, growth hormone deficiency may occur, leading to growth retardation. Renal manifestations include proteinuria, focal segmental glomerulosclerosis, and Fanconi tubular disease. The hematological manifestations are anemia and lactic acidosis. There are also muscle manifestations such as exercise intolerance and proximal muscle weakness. ME patients may experience gastrointestinal symptoms (Chinese Medical Association Neurology Branch, 2015; Mancuso et al., 2014; Wang et al., 2008), such as constipation, diarrhea, gastric motility disorders, CIPO, recurrent or periodic vomiting, and recurrent pancreatitis. The patient experienced abdominal distention in 2007. The patient’s previous diabetes, hearing loss and chronic abdominal distention may be the early initial manifestations of MELAS, which was not considered. In 2022, severe recurrent intestinal obstruction began to appear.

### 3.4 Treatment of MEALS

Currently, there is no specific treatment for MELAS, and long-term use of antioxidants and supplemental factors is needed for MELAS treatment (Yan et al., 2024). However, there is no consensus on the acute treatment or prevention of stroke like attacks. Some centers use l-arginine, l-citrulline, taurine, l-carnitine, and coenzyme Q10 to treat stroke like attacks, but the evidence base is weak (Barcelos et al., 2020). The current clinical focus is on treating neurological, cognitive, metabolic, musculoskeletal, gastrointestinal, and cardiopulmonary complications. The current treatment mainly targets regulating mitochondrial autophagy, increasing mitochondrial biosynthesis, and restoring the production of nitric oxide and nucleotide library. Recently, new therapeutic methods such as interventions to regulate mitochondrial genome gene expression, solid organ transplantation, and stem cell transplantation have emerged. Nutrients such as branched chain amino acids, glutamine, arginine, nucleotides, and antioxidants can regulate the immune system related to inflammation and metabolic pathways, known as “immunonutrition”, many of which have been used for the treatment of MELAS patients (Pérez-Cruz et al., 2022).

It should also be noted that some drugs with mitochondrial toxicity, such as valproic acid, can inhibit fatty acid oxidation, tricarboxylic acid cycle and oxidative phosphorylation (Silva et al., 2008). Similarly, phenytoin, carbamazepine and phenobarbital also have mitochondrial toxicity, so it is not suitable for MELAS patients to use these anticonvulsants to control epilepsy. On the contrary, levetiracetam tablets and wireless particle toxicity can be used for MELAS patients. Metformin can inhibit oxidative phosphorylation, enhance glycolysis, and lead to lactic acidosis. Therefore, diabetic patients in MELAS should avoid using metformin as much as possible (Parikh et al., 2009). The mitochondrial toxicity of olanzapine is controversial. Studies have shown that olanzapine can induce mitochondrial division (Del Campo et al., 2018) reduce mitochondrial enzyme activity and ATP synthesis in leukocytes, which indicates that olanzapine has certain mitochondrial toxicity (Eftekhari et al., 2016). Some studies on mitochondrial toxicity have shown that olanzapine has minimal or no direct mitochondrial toxicity. When olanzapine is used to treat the mental symptoms of patients with MELAS, it is necessary to evaluate and pay close attention to the reaction after medication (Kramar et al., 2024).

### 3.5 Clinical manifestations and pathological mechanisms of CIPO

The gastrointestinal manifestations of MELAS patients are rarely documented in the literature (Hom and Lavine, 2004). The reported symptoms include difficulty swallowing, abdominal pain, bloating, diarrhea, constipation, and gastroparesis. Severe cases may progress to CIPO (Narbonne et al., 2004; de Laat et al., 2015; Sproule and Kaufmann, 2008), often leading to severe weight loss or cachexia. However, the mechanism by which MD causes gastrointestinal dysfunction is still unclear (Koga et al., 2006). CIPO is a disease with symptoms and signs similar to those of mechanical intestinal obstruction but without any underlying cause of mechanical obstruction. It is a rare chronic intestinal obstruction syndrome that occurs in the absence of any obvious organic lesions blocking the intestinal lumen and is characterized by episodic exacerbation (Jinnouchi et al., 2022). CIPO can be classified as primary or secondary, with MD being the most common cause of secondary CIPO. There are currently two possible pathological mechanisms for intestinal pseudo-obstruction: one is the origin of smooth muscle. In MD carrying mitochondrial DNA 343A>G mutations, severe deficiency of cytochrome C oxidase in the gastrointestinal tract leads to dysfunction of the smooth muscle cell respiratory chain. Another type is derived from vascular endothelial cells (Wu et al., 2020). In MELAS patients, endothelial dysfunction leads to a reduced vasodilation ability of small arteries during the attack period, and patients carrying mitochondrial DNA 343A>G mutations may also develop ischaemic colitis (Hess et al., 1995). A comprehensive pathological analysis of autopsy materials from a 52-year-old male with MELAS (m.3243A>G mutation) and recurrent symptoms of intestinal obstruction revealed extensive vacuolization and pallor in the smooth muscles of the entire gastrointestinal tract. Electron microscopy revealed abundant aberrant mitochondria in smooth muscle cells (Kawano et al., 2024), whereas muscle filaments were significantly lost in colon muscles. These findings suggest that mitochondrial dysfunction may directly impair smooth muscle contraction (Nagao et al., 2026).

### 3.6 Diagnosis and differential diagnosis of CIPO

Ohkubo et al. proposed the following diagnostic criteria for CIPO: a. presence of one or more intestinal obstruction symptoms at least 6 months prior to diagnosis; b. bloating, abdominal pain or both symptoms in the past 12 weeks; c. abdominal X-ray, ultrasound, and/or CT imaging showing intestinal dilation and/or gas-liquid levels; and d. no evidence of structural disease (Ohkubo et al., 2012). In the abdominal CT scan performed on the patient, small intestine dilation and gas-liquid levels were observed without any mechanical obstruction, accompanied by years of recurrent abdominal distension and constipation. Therefore, the patient met the diagnosis of CIPO. This patient needs to be differentiated from Superior Mesenteric Artery Syndrome (SMAS), secondary CIPO from diabetes autonomic neuropathy or diabetes gastroparesis (Jinnouchi et al., 2022), Mitochondrial Neurogastrointestinal Encephalomyopathy (MNGIE). SMAS is also a rare disease characterized by gastrointestinal symptoms caused by compression of blood vessels at the level of the duodenum at the angle between the aorta and the superior mesenteric artery. It is typically associated with reduced fat mass in the mesentery and retroperitoneum, as well as several triggering factors leading to weight loss. Diabetes gastroparesis has been observed mainly in patients with diabetes for a long period of time. It is a symptom of diabetes autonomic neuropathy, accompanied by nausea, abdominal distention, anorexia, postmeal vomiting and other symptoms and signs. MNGIE is an autosomal recessive genetic disease caused by a mutation in the 22q13.32 gene. Common clinical manifestations include unexplained weight loss, emaciation, and cachexia under normal diet and nutrient intake; Symptoms of the digestive system such as nausea, vomiting, severe abdominal pain, bloating, difficulty swallowing, constipation and diarrhea, diverticulitis of the small intestine, and dilation of the stomach and intestines; Progressive abduction nerve paralysis, ptosis, polyneuropathy, hearing loss and other neurological symptoms; Imaging manifestations of white matter encephalopathy; Fatty liver is accompanied by cirrhosis, pancreatitis, early diabetes, elevated triglycerides, elevated blood lactate and other metabolic abnormalities (Maev et al., 2022). SMAS and diabetes gastroparesis can be excluded because the patient’s abdominal CT does not reveal vascular compression, and the patient’s blood sugar level basically reaches the standard. MNGIE can also exclude gene mutation sites that are inconsistent with MELAS. Moreover, MNGIE follows an autosomal recessive inheritance pattern rather than matrilineal inheritance.

### 3.7 Treatment of CIPO

The treatment goal of CIPO is to relieve symptoms, enhance intestinal motility, improve malnutrition, and reduce complications as much as possible. It usually needs multidisciplinary cooperation, including nutritional support, drug therapy, endoscopic intervention and surgery. There are many causes of secondary CIPO, so it is necessary to make individualized plans according to the specific causes (Peng and Zuo, 2025).

Nutritional support is the basis of CIPO treatment (Benjamin et al., 2010). For patients who can take oral diet, encourage patients to take small amounts of food many times, eat low-fat, low fiber diet, and avoid eating fermented food (De Giorgio et al., 2011; Gabbard and Lacy, 2013; Billiauws et al., 2014; Kirby et al., 2018). Patients who cannot eat by mouth need enteral nutrition support (Zenzeri et al., 2020).

Drug treatment can also be carried out. The drug treatment of secondary CIPO varies according to the primary disease. The purpose of treatment is to control the progress of primary disease, increase gastrointestinal motility, inhibit bacterial growth, improve intestinal inflammation, and reduce abdominal pain symptoms. However, the curative effect of drug therapy is still uncertain. Experimental administration can be used to determine which drug patients are sensitive to. Metoclopramide and domperidone are dopamine D2 receptor antagonists, which need to monitor the QT interval of ECG (Camilleri et al., 2013). Erythromycin and azithromycin are macrolide antibiotics, which mainly act on motilin receptors in the upper gastrointestinal tract and promote gastric emptying. Low dose erythromycin can act on the motilin receptor in the upper gastrointestinal tract to promote gastric emptying. It can be used in patients with upper gastrointestinal symptoms to reduce the severity and frequency of obstructive symptoms. However, erythromycin has limited therapeutic effect on patients with neurogenic CIPO and severe gastrointestinal motility disorders (Downes et al., 2018; Emmanuel et al., 2004; Chini et al., 2012; Dalgic et al., 2005). Octreotide is a long-acting somatostatin analogue, which can induce phase III transitional complex movement in the small intestine, reduce the secretion of digestive juice and reduce the gastrointestinal burden, but it may also cause the inhibition of gastric antrum peristalsis, which may induce or aggravate the symptoms of gastroparesis, especially after meals. Therefore, octreotide is recommended to be used at night (Edmunds et al., 1998). Anticholinesterase inhibitors, such as neostigmine and pyridostigmine, can increase cholinergic synaptic transmission and promote exercise. Neostigmine is a short-acting anticholinesterase inhibitor, which is usually used in patients who are ineffective in other non-surgical treatment (Odea et al., 2009). Acotiamide is an acetylcholinesterase inhibitor, which can enhance the effect of acetylcholine on the intestinal nervous system, and plays an important role in the treatment of functional dyspepsia (Ikeo et al., 2017). Acotiamide can enhance the role of cholinergic nerve in the stomach, promote the motility of gastric antrum and improve gastroparesis (Matsueda et al., 2010). Acotiamide can inhibit M1 and M2 muscarinic receptors, inhibit acetylcholinesterase, and enhance the release of acetylcholine in the stomach (Ogishima et al., 2000; Horná et al., 2024). Procapride is a highly selective 5-HT4 agonist, which can increase the gastrointestinal motility and improve the gastrointestinal function of CIPO patients (Valle and Godoy, 2014). Bacterial overgrowth in the small intestine can lead to inflammation of the intestinal mucosa and further damage the intestinal motility, which is one of the complications of CIPO. European clinical nutrition and metabolism guidelines recommend that adult CIPO should use periodic and cyclic antibiotics to prevent bacterial overgrowth in the small intestine, and non absorbable antibiotics such as rifaximin and aminoglycosides should be preferred. However, it should be noted that aminoglycosides antibiotics can cause mtDNA translation damage and have certain mitochondrial toxicity, which should be evaluated before use (Parikh et al., 2009). And some broad-spectrum antibiotics such as amoxicillin, clavulanic acid, metronidazole, quinolones, doxycycline and antifungal compounds (Thapar et al., 2018). Similarly, chloramphenicol and linezolid also have mitochondrial toxicity, so it should be avoided (Gopan and Sarma, 2021). Intestinal microecological therapy includes enteral nutrition, probiotics, prebiotics and fecal bacteria transplantation, which may bring benefits to CIPO patients.

Endoscopic treatment and surgery in CIPO treatment are mainly used to relieve the symptoms of obstruction caused by the expanded gastrointestinal segment, while maintaining the nutritional status. Intestinal transplantation can be used to treat CIPO patients with ineffective drug treatment or severe complications of HPN. There are also emerging treatment methods such as intestinal microecology therapy and electrical stimulation therapy (Peng and Zuo, 2025). The drug treatment of secondary CIPO varies according to the primary disease. CIPO secondary to MELAS needs multidisciplinary management. In the acute phase, it needs the cooperation of the Department of nutrition in the Department of Neurology and gastrointestinal surgery, and can be treated in a multidisciplinary palliative treatment team that can provide comprehensive treatment.

### 3.8 Research on complications of other systems in ME

The literatures on ME and other systemic complications except MELAS and gastrointestinal complications were retrieved on PubMed for review. The incidence, mechanism and clinical managers of complications in different systems of ME have been briefly summarized into a table (Table 1).

**TABLE 1:** The incidence, mechanism and clinical managers of complications in different systems of ME.

MELAS complications	Incidence	Mechanism	Clinical management
Fever and headache	10%	May be related to infection and epileptic seizures, or possibly due to lesion involvement in the hypothalamic fever center	Avoiding misdiagnosis as herpes simplex encephalitis
Heart disease companion	38%	Mitochondrial dysfunction leads to energy metabolism tending towards glycolysis, lactate accumulation in multiple organs, and myocardial hypertrophy may be an early compensatory response to cardiac disease. Pathological changes include interstitial fibrosis and myocardial fiber hypertrophy	MELAS requires cardiac examination. Carry out symptomatic drug treatment to reduce ventricular hypertrophy and heart failure
Diabetes and deafness	No data found	Mitochondrial dysfunction can lead to the function of foreign trade cells and pancreatic β cells	Timely identify the cause of MD and give corresponding treatment
Eye symptoms	No data found	Failure of melanin granules in retinal pigment epithelial cells	Identifying different retinopathy is helpful to distinguish different MD
Changes in mental state	No data found	Possible risk factors include disease duration, gastrointestinal damage, and epilepsy	The society and family need to give physical care and emotional care to patients with MELAS
Kidney disease	Low	The renal lesions associated with m.3243a > g usually showed subnephrotic proteinuria and gradual deterioration of renal function	Organ transplantation is one of the methods to treat MD related nephropathy

#### 3.8.1 Fever and headache

A total of 8 results of “mitochondrial encephalomyopathy, fever, headache” were retrieved from PubMed. After excluding 2 articles on neuronal inclusion body disease, there were 6 results.

Some literature reports that certain ME present with fever and headache as initial symptoms, often misdiagnosed as viral encephalitis (Peng et al., 2019). The first symptom of MELAS is fever, which is only 10% (El-Hattab et al., 2015), but 90% of patients with herpes simplex encephalitis will be found (Levitz, 1998), this leads to a high probability of misdiagnosis as encephalitis, which can be identified by the number of white blood cells in cerebrospinal fluid and gene second-generation sequencing (Zeng et al., 2022). The mechanism of fever in MELAS may be related to infection and epileptic seizures, or possibly due to lesion involvement in the hypothalamic fever center (Wang and Kong, 2017; Zhang et al., 2019; Chen et al., 2018; Chen et al., 2016; Chen et al., 2007; Sun et al., 2010).

#### 3.8.2 Heart disease companion

A total of 204 results were obtained by searching for “mitochondrial encephalomyopathy, heart” on PubMed, excluding 84 articles unrelated to heart disease, with a total of 120 results.

The heart is also one of the organs susceptible to mitochondrial disease due to its vigorous energy metabolism. 38% of MELAS patients will have heart disease (Fayssoil, 2009). MELAS patients may develop various types of cardiac lesions, including hypertrophic cardiomyopathy and dilated cardiomyopathy (Majamaa-Voltti et al., 2002), heart conduction block, heart failure, etc. Simple mitochondrial cardiomyopathy is rare and often a manifestation of systemic mitochondrial disease. Its mechanism of action is that mitochondrial dysfunction leads to energy metabolism tending towards glycolysis, lactate accumulation in multiple organs, and myocardial hypertrophy may be an early compensatory response to cardiac disease. Pathological changes include interstitial fibrosis and myocardial fiber hypertrophy (Brambilla et al., 2019). The typical symptoms of mitochondrial disease do not appear until 30 years after the onset of cardiac symptoms, so mitochondrial disease cardiac lesions require long-term follow-up observation (Zhao et al., 2022). Studies have found that 40% of MELAS patients were diagnosed with left ventricular hypertrophy by echocardiography (Anan et al., 1995), and 56% of 3243a > g mutation patients had left ventricular hypertrophy, and the degree of mutation was linearly correlated with left ventricular mass index (Majamaa-Voltti et al., 2002). MELAS may be associated with pulmonary hypertension (Sproule et al., 2008). MELAS complicated with cardiomyopathy will lead to poor prognosis. Some studies have shown that the survival rate of children with MELAS complicated with cardiomyopathy at the age of 16 is 18% (while the survival rate of children without MELAS complicated with cardiomyopathy is 95%) (Sca et al., 2004). MELAS patients may have serious cardiac complications, such as malignant arrhythmia, heart failure and sudden death (Finsterer and Kothari, 2014; Fayssoil, 2009; Silvestri et al., 1997). The cardiac biopsy of MELAS patients with cardiomyopathy showed that the myocytes in the myocardium were unevenly distributed and abnormally thickened. Myocardial electron microscopy showed that the number of mitochondria increased significantly, and abnormal cristae and myofibrils decreased. Many mitochondria have sub crystals and lipid inclusions, which are the characteristics of mitochondrial cardiomyopathy. In the heart of MELAS patients with cardiomyopathy, the number of abnormal mitochondria in myocardial cells increased significantly. The expression levels of atrial natriuretic peptide, B-type natriuretic peptide, α - skeletal muscle actin and β - myosin heavy chain were significantly increased in the left and right ventricles of MELAS patients with cardiomyopathy (Hsu et al., 2016). Therefore, it can be concluded that patients with MELAS need cardiac examination, including invasive cardiac and/or skeletal muscle biopsy, 12 lead ECG and Holter monitoring, cardiac MRI and echocardiography to assess the degree and type of myocardial remodeling (Bates et al., 2012). At present, there is no specific treatment for patients with MELAS complicated with cardiomyopathy, but symptomatic treatment, such as β - blockers, angiotensin converting enzyme inhibitors and angiotensin receptor blockers, can reduce left ventricular hypertrophy (Yancy et al., 2013). Aldosterone receptor antagonists such as spironolactone and eplerenone can treat systolic heart failure (Pitt et al., 1999; Zannad et al., 2011). Defibrillator and cardiac resynchronization therapy can also be used, and heart transplantation has also been successfully implemented (Bonnet et al., 2001; Tranchant et al., 1993; Schmauss et al., 2007).

#### 3.8.3 Diabetes and deafness

A total of 371 results were obtained by searching for “mitochondrial encephalomyopathy, diabetes” on PubMed, excluding 152 articles unrelated to diabetes, with a total of 219 results. A total of 222 results were obtained by searching for “mitochondrial encephalomyopathy, deafness” on PubMed, excluding 100 articles unrelated to deafness, with a total of 122 results.

MELAS also has a special type, maternal inherited diabetes with deafness syndrome, which is characterized by early onset age, emaciation, maternal genetic history, fasting low C-peptide, hearing loss, etc (Ge et al., 2022) Outer hair cells in the cochlea have metabolically active and highly energy dependent characteristics. Mitochondrial dysfunction can cause a decrease in ATP levels, which in turn can lead to a decline in the function of supplying outer hair cells and cause hearing loss (Chen et al., 2025). The metabolism of pancreatic β cells is active, and mitochondria play a huge role. Mitochondrial dysfunction will hinder the insulin secretion of pancreatic β cells, aggravate the apoptosis of pancreatic β cells, and destroy the glucose homeostasis. Mitochondrial damage will lead to the aggravation of oxidative stress, which will affect the insulin signaling pathway and lead to glucose imbalance, which will lead to the aggravation of insulin resistance and prone to diabetic complications (Qin et al., 2025). Worldwide, there are many patients with diabetes and hearing loss. It is necessary to timely identify the cause, especially MD, and not only symptomatic treatment, but also timely cause treatment.

#### 3.8.4 Eye symptoms

A total of 102 results were obtained by searching for “mitochondrial encephalomyopathy, eye” on PubMed, excluding 36 articles unrelated to eye symptoms, with a total of 66 results.

The ocular manifestations of MELAS include macular degeneration, abductor ophthalmoplegia, blepharoptosis and posterior subcapsular cataract, as well as pigmented retinopathy, abductor ophthalmoplegia, blepharoptosis and posterior subcapsular cataract (Liu et al., 2025; Na and Lee, 2024; De Laat et al., 2013). Retinal diseases caused by mitochondrial DNA deletion can be divided into three types based on retinal imaging: type 1 is mild, focal Pigment Abnormalities; Type 2 is multifocal white yellow subretinal deposition and pigment changes, limited to the posterior pole; Type 3 showed extensive granular pigment changes. The late stage of type 2 and type 3 retinopathy is chorioretinal atrophy, which usually occurs in the optic papilla and surrounding areas, but does not affect the macular area. The clinical manifestations of retinopathy caused by different types of MD are different. Identifying different retinopathy is helpful to identify different MD (Birtel et al., 2022). The MD of m.3243G>A gene mutation is divided into four grades according to the severity, ranging from mild abnormal pigmentation to chorioretinal atrophy of macula. The fundus changes related to m.3243G>A gene mutation usually only appear in the posterior pole of the retina, and the response of full field electrophysiological examination is normal (De Laat et al., 2013; Massin et al., 1999). Histological studies have shown that mitochondrial retinopathy leads to the depletion of melanin granules in retinal pigment epithelial cells.

#### 3.8.5 Other atypical onset symptoms

There are also changes in mental state, kidney disease, blood disease, muscle weakness, growth hormone deficiency, etc. MELAS also has other atypical onset symptoms, such as acute respiratory failure and conduction aphasia, etc (Fan et al., 2020) But these complications are rarely reported.

Some MELAS patients are prone to depression, which can lead to a decrease in their quality of life. Possible risk factors include disease duration, gastrointestinal damage, and epilepsy (Wei et al., 2017). In another case, mental symptoms are the early manifestations of me, including auditory hallucinations, delusional emotions, psychomotor excitement and anxiety or anxiety, but there is no obvious disturbance of consciousness (Suzuki et al., 1989). The society and family need to give physical care and emotional care to patients with MELAS, which can reduce the depression of patients with MELAS. The patient had psychotic symptoms of aggressive behavior, which improved after olanzapine treatment.

Hereditary mitochondrial defects are rare in pediatric and adult patients with renal damage (Hall and Unwin, 2007). m.3243A>G gene mutation is one of the most common gene mutations associated with kidney disease. In MD, there is usually no hematuria and no destruction of glomerular basement membrane. The renal lesions associated with m.3243A>G usually showed subnephrotic proteinuria and gradual deterioration of renal function (Liu et al., 2025). Organ transplantation is one of the methods to treat MD related nephropathy (Lim et al., 2017).

### 3.9 DNER positive

The patient’s serum is positive for DNER with a titer of 1:30. DNER is an autoantibody against Purkinje cells in the cerebellum, and the core associated disease is paraneoplastic cerebellar degeneration: most cases are severe cerebellar damage caused by distant effects of Hodgkin lymphoma. The clinical manifestation is that patients will rapidly develop severe cerebellar ataxia, characterized by unstable walking (gait abnormalities), limb tremors, unclear speech (articulation disorders), and nystagmus. Neurological symptoms often appear months or even years before lymphoma is detected. Although the patient tested positive for DNER, no corresponding clinical manifestations were observed. I have been in contact with the patient’s family until now, and the patient did not show such symptoms during follow-up. The lymph node ultrasound examination performed on 30 November 2022 showed no abnormalities. Therefore, considering the DNER positive result is accidental, as the patient did not have a high suspicion of Hodgkin’s lymphoma and did not undergo further cerebrospinal fluid DNER examination or other related tests for Hodgkin’s lymphoma.

## 4 Conclusion

In summary, this patient is a rare case of late-onset MELAS with recurrent CIPO. Currently, the majority of published MELAS cases are in adolescents, with very few cases occurring in elderly individuals. The later the onset age is, the lower the proportion of m.3243A>G mutations detected in the blood, which is not related to the severity of the disease. The lack of muscle biopsy and small intestine pathological biopsy is a limitation of this case, as the patient’s family refused to undergo invasive examinations. According to the mutation in the m.3243A>G gene, there is no doubt about the diagnosis of MELAS based on the clinical and imaging manifestations of stroke like seizures, epileptic seizures, and lactic acidosis in the patient. During acute attacks of intestinal obstruction, the patient experienced intestinal torsion. During open abdominal exploration, only the twisted small intestine was repositioned without undergoing resection and sampling for pathological biopsy. However, based on the patient’s clinical manifestations of chronic recurrent abdominal distension and constipation, abdominal CT showed signs of intestinal dilation and intestinal obstruction, which supports the diagnosis of CIPO. Although there is no muscle biopsy or intestinal tissue pathological biopsy, it does not affect our inference of MELAS and CIPO diagnosis. Although MELAS has been well controlled through drug treatment for stroke, such as seizures, epileptic seizures, and lactic acidosis, CIPO occurs earlier and recurs, showing a gradually worsening trend. If the cause of CIPO can be identified early and treated appropriately to avoid the use of drugs with mitochondrial toxicity that further worsen the condition, it can delay the course of the disease and prolong the patient’s life. Not only CIPO, but also all systemic complications of MELAS need to be understood clinically. Patients suspected of having MD are recommended to undergo relevant examinations as soon as possible, especially genetic testing. However, there is currently no specific treatment for CIPO caused by MELAS. This study aims to increase the attention of medical researchers to this disease and invest more research into the treatment of the disease to reduce the degree of pain experienced by patients.
