# Correction: Late-onset mitochondrial encephalomyopathy lactic acidosis and stroke-like episodes with chronic intestinal pseudo-obstruction: a case-based review

**DOI:** 10.3389/fmolb.2026.1988307

**Published:** 2026-10-14

**Authors:** Lin Zhang, Junjian Lin, Fengying Li, Yujie Jia, Junhua Li, Xiaopeng Wang

**Affiliations:** 1 Department of Neurology, Rizhao Central Hospital, Rizhao, China; 2 Department of Rehabilitation Medicine, Rizhao Central Hospital, Rizhao, China; 3 Department of Neurology, The Second Hospital of Hebei Medical University, Shijiazhuang, China

**Keywords:** mitochondrial encephalomyopathy, mitochondrial encephalomyopathy lactic acidosis and stroke-like episodes, chronic intestinal pseudo-obstruction, m.3243A>G gene mutation, late-onset

Affiliation Department of Rehabilitation Medicine, Rizhao Central Hospital, Rizhao, China was erroneously omitted for author Yujie Jia. Affiliation Department of Neurology, The Second Hospital of Hebei Medical University, Shijiazhuang, China was erroneously omitted for author Xiaopeng Wang.

Affiliation Department of Neurology, Rizhao Central Hospital, Rizhao, China was erroneously included in the paper. This affiliation has now been removed for author Yujie Jia. Affiliation Department of Neurology, Second Hospital of Hebei Medical University, Shijiazhuang, China was erroneously included in the paper. This affiliation has now been removed for author Xiaopeng Wang.

The original version of this article has been updated.

